# An analysis of threats, strategies, and opportunities for African rhinoceros conservation

**DOI:** 10.1002/ece3.7536

**Published:** 2021-05-01

**Authors:** Admire Chanyandura, Victor K. Muposhi, Edson Gandiwa, Never Muboko

**Affiliations:** ^1^ School of Wildlife, Ecology and Conservation Chinhoyi University of Technology Chinhoyi Zimbabwe; ^2^ Scientific Services Zimbabwe Parks and Wildlife Management Authority Harare Zimbabwe

**Keywords:** COVID‐19 pandemic, external shock, poaching, wildlife forensics, wildlife trade and trafficking

## Abstract

The complexity and magnitude of threats to black (*Diceros bicornis*) and white (*Ceratotherium simum*) rhinoceros conservation in Africa have triggered global concerns and actions. In this study, we analyzed (i) threats to rhinoceros conservation including external shocks, (ii) historical rhinoceros conservation strategies in Zimbabwe and Africa, more broadly, and (iii) opportunities for enhanced rhinoceros conservation in Zimbabwe and Africa. A literature search from 1975 to 2020 was carried out using a predefined search protocol, involving a number of filters based on a set of keywords to balance search sensitivity with specificity. A total of 193 articles, which were most relevant to key themes on rhinoceros conservation, were used in this study. The common threats to rhinoceros conservation identified in this paper include poaching, habitat fragmentation and loss, international trade in illegal rhino products, and external shocks such as global financial recessions and pandemics. Cascading effects emanating from these threats include small and isolated populations, which are prone to genetic, demographic, and environmental uncertainties. Rhinoceros conservation strategies being implemented include education and awareness campaigns, better equipped and more antipoaching efforts, use of innovative systems and technologies, dehorning, and enhancing safety nets, and livelihoods of local communities. Opportunities for rhinoceros conservation vary across the spatial scale, and these include (a) a well‐coordinated stakeholder and community involvement, (b) strategic meta‐population management, (c) enhancing law enforcement initiatives through incorporating real‐time surveillance technologies and intruder detection sensor networks for crime detection, (d) scaling up demand reduction awareness campaigns, and (e) developing more certified wildlife crime and forensic laboratories, and information repository for international corporation.

## Graphical abstract

African rhinoceros conservation is facing serious threats, which require clear strategies and using all opportunities to combat them.

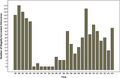

## 1 INTRODUCTION

A global unprecedented loss of wild species associated with high rates of extinction has been recently reported (Bongaarts, 2019; Brondizio et al., 2019; Johnson et al., 2017; Quesada, 2019). This extinction crisis prompted participants at the Society for Conservation Biology Conference (Stoett, 2019) in Kuala Lumpur to declare it “The Crisis of Humanity.” Substantial efforts are being made to up‐scale global conservation efforts, and decision support frameworks are being put in place to reduce the loss of wildlife species in their native range states (Ceballos et al., 2017; Schwartz et al., 2018; Travers et al., 2019). Concern over the loss of large and charismatic mammalian species, for example, African elephant (*Loxodonta africana*), and black (*Diceros bicornis*) (Amin et al., 2006; Emslie, 2020b) and white (*Ceratotherium simum*) (Emslie, 2020a; Harper et al., 2013) rhinoceros in Africa, has been raised (Emslie et al., 2016; Grooten & Almond, 2018). The main threats responsible for global species loss include increased poaching pressure and illegal wildlife trade (Atkins et al., 2018; Challender & MacMillan, 2014; Herbig & Minnaar, 2019) and habitat loss and fragmentation due to increasing human population pressure (Cheteni, 2014; Fahrig et al., 2019; Fletcher et al., 2018). Two rhinoceros subspecies in sub‐Saharan Africa, the black and white rhinoceros, are classified by the International Union for the Conservation of Nature (IUCN) as critically endangered and near threatened, respectively (Emslie, 2020a,2020b; Janssens & Trouwborst, 2018). These two species were once abundant and widely distributed in the sub‐Saharan region (Kagande & Musarurwa, 2014; Rosen & Smith, 2010), but their abundance and range of occurrence have since been significantly reduced (Jill et al., 2018). Long histories of illegal harvesting and international trade in black and white rhinoceros horn compounded by weak law enforcement are some of the main factors leading to the decline in these species populations (Leader‐Williams, 1992; Milner‐Gulland et al., 1992; Rubino & Pienaar, 2017). The cascading effects of illegal harvesting and trade, combined with habitat fragmentation and loss, have led to small isolated rhinoceros populations (Fox et al., 2017; Hoffmann et al., 2015; Singh et al., 2017). The persistence of these small populations in the wild is compromised due to random chance events, that is, demographic, environmental, and genetic stochasticity processes (Castorani et al., 2017; Kundu et al., 2017; Lacy, 2019). These stochastic processes tend to increase the probability of extinction of small populations in wild in the absence of strategic conservation measures (Adams & Makramalla, 2015; Comino & Ferretti, 2016; Jill et al., 2018; Whitehead et al., 2017).

The coronavirus disease 2019 (COVID‐19) pandemic is the latest episode in a string of environment‐borne human tragedies, catastrophic in its magnitude, reach, and repercussions (Bang & Khadakkar, 2020). Besides threatening millions of human lives, devastating social and economic conditions globally, the COVID‐19 pandemic poses a challenge to the protection of Africa's iconic wildlife species, particularly fragile populations (Ahmand et al., 2020; Roth, 2020) such as black and white rhinoceros. COVID‐19 pandemic has affected law enforcement in most protected areas due to reduced capacity by park staff (Beirne, 2020; Corlett et al., 2020). It is envisaged that there will be a surge in poaching as observed in some areas where communities resort to illegal hunting of wildlife for a means of survival under COVID‐19 pandemic‐related lockdown circumstances (Neupane, 2020). Critical wildlife conservation programs are facing funding cuts due to revenue shortfalls occasioned by COVID‐19 pandemic disruptions and travel restrictions, which affect mobility of potential tourists (Zela, 2020). The drop in ecotourism activities reduces revenue generation and financial resources meant for wildlife conservation (Lindsey et al., 2020; Neupane, 2020; Waithaka, 2020). COVID‐19 pandemic also presents a very difficult puzzle for the wildlife‐reliant communities, particularly on conservation work. Currently, most governments and nongovernmental agencies have prioritized controlling COVID‐19 pandemic over supporting wildlife protection (Lendelvo et al., 2020; Lindsey et al., 2020). Anthropogenic pressures are becoming widespread owing to the mass urban–rural migration and unemployment in most biodiversity‐rich countries in Africa. Consequently, the species and habitats of concern may be in danger of anthropogenic disturbances such as poaching, mining, logging, and diseases (de Almeida‐Rocha et al., 2017; Fischer & Lindenmayer, 2007).

International efforts focused on the Convention on International Trade in Endangered Species (CITES), which enacted an international ban on the trade of white rhinoceros parts at its first conference of the parties in 1975 (CITES, 2013; Emslie et al., 2016; Hutton & Dickson, 2000; Martin, 2013). Rhinoceros and rhinoceros parts were among the first species to be included on the CITES Appendices (De Alessi, 2000; Leader‐Williams, 1992; Milner‐Gulland et al., 1992). In 1975, the black rhinoceros was placed in Appendix II. In 1977, both the black and southern white rhinoceros were placed in Appendix I (Leader‐Williams, 1992; Milner‐Gulland et al., 1992; Warmenbol & Smith, 2016). The CITES trade ban on global rhinoceros horn trade has created a significant market failure that jeopardizes rhinoceros conservation on private lands by limiting legal revenues from rhinoceros (Codron et al., 2007; Rubino & Pienaar, 2017). The CITES trade ban has, however, not halted or reduced rhinoceros losses to poaching, and it does not seem to stop the trade in rhinoceros horn (Table 1). If anything, the Appendix I listings led to a sharp increase in the black market price of rhinoceros horn, which simply fueled further poaching and encouraged speculative stockpiling of horn (Martin, 2013).

**TABLE 1:** Selected African rhinoceros conservation initiatives 1975–2016

Year	Key initiative	Coverage or level of application	Approach	Impact of rhinoceros conservation initiative	References
1975	Convention on International Trade in Endangered Species of Wild Fauna and Flora (CITES)	Global	Regulated rhinoceros trading	Failed to stop the illegal trade in rhinoceros horn rather fueled the illegal trade and caused rhino horn price to rise dramatically. Between 1970 and 1994, black rhinos suffered a 95% decline. Created a significant market failure and limited legal revenues. Black rhinoceros went extinct within at least 18 range states in Africa. CITES has been highly successful in providing the most comprehensive database on international trade in wildlife species available to date	Leader‐Williams (1992) De Alessi (2000) Rubino and Pienaar (2017) Tietenberg and Lewis (2009) IUCN (1988)
1985	Operation Stronghold	Zimbabwe	Patrols by armed game scouts Harare‐based Park authorities, white‐controlled An antipoaching approach	Antipoaching units intercepted and stopped poachers especially from Zambia but rhino populations kept dwindling in Zambezi Valley Failed to ameliorate the culture of hostility created by colonial land and resource alienation. Colonial metaphor, rather than trust, was still very strong. Formed a rift between conservation units and local people. Communities had no economic incentive in helping to protect rhinos	Hill (1991) Wildlife (1989), Zimbabwe Wildlife (1989, 2018)
1989	Convention on International Trade in Endangered Species of Wild Fauna and Flora	Global	International Trade in Ivory ban	Dramatic fall in poaching levels for the immediate few years afterward Few years later poaching increased CITES has been very successful in providing the most comprehensive database on international trade in wildlife species available to date. It later gave rise to illegal markets and increased illegal harvesting. Burgeoning market demand	Hutton and Dickson (2000) Leader‐Williams (1992) du Toit (2002) Martin (2013) Rubino and Pienaar (2017)
1989	Communal Areas Management Program for Indigenous Resources (CAMPFIRE)	Zimbabwe	Integrated conservation and development projects (ICDPs) Sustainable wildlife utilization scheme	Broaden local ownership and management of wildlife and other natural resources. Decline in human–wildlife conflicts. Local people benefited directly from conserving wildlife. Rhinoceros in protected areas adjacent to CAMPFIRE communities were considered to be safe.	Martin (1993) Metcalfe (1993) Murphree (2009) Madzudzo (1997)
1991	Conservancy Project (CP)	Southern African Development Community (SADC)	Change in land use from cattle ranching to wildlife ranching	A robust and diversified wildlife sector Extensive multispecies production systems Ecological success Reintroductions improved the genetics of rhinoceros. Remarkable population increases	du Toit (1994) Alibhai and Jewell (1994, 1994) Wolmer (2005) Du Toit (2006)
1992	Southern African Development Community Rhino Management Group (SADCRMG)	SADC	Guidelines for implementing SADC rhino conservation strategies	Maintenance of viable and well‐distributed meta‐population of Southern African rhino taxa focus on promoting and implementing a regional strategy. SADC development policies Maintain accurate population estimates and demographic measures	Leader‐Williams (1992) du Toit (1994) Emslie (2013) Du Toit (2006), Emslie et al. (2009)
	Intensive protection Zone (IPZ)	Zimbabwe	Organized and Intensive rhinoceros protection	Black rhino increase from 300 individuals to 400 between 1994 and 1998 Poaching numbers declined soon after IPZ formation Improvement was noticeable, but later failed to address the corruption issue or to adequately protect the remaining rhinos Corruption, lack of financial resources, and demoralized staff hampered the execution of IPZ duties	Miliken et al. (1993) De Alessi (2000) Costello et al. (2012)
1992	Rhinoceros Dehorning	Africa	Crisis antipoaching measure Deterrent to poaching	Deterred poachers and rapid horn regrowth Displaced the crime Effective in reducing rhino mortality Mortality rates are now less than 2 %Poaching incidences reduced significantly Increased the value of illegally held stockpiles	Emslie and Brooks (1999) Lindsey and Taylor (2011), Milner‐Gulland et al. (1992), Milner‐Gulland et al. (1994) Emslie et al. (2009)
	SADC Rhinoceros Program (SADCRP)	SADC	Implement a pragmatic regional rhino strategy within the SADC region	Logical approach of harvesting rhinos steadily Meta‐population growth Well‐organized translocations Professional monitoring, training, and development	Cumming et al. (1990) Cunningham et al. (1999) Emslie and Brooks (1999) De Alessi (2000) du Toit (2002)
	Black Rhino Conservation Strategy	Zimbabwe	Coordination, collaboration and program management	Led to the establishment of and operations within Intensive Protection Zones (IPZs) Cases of black rhino poaching were reported and the population grew at a rate of 10% per annum Appropriate management actions Enhance incentives for rhino conservation	1997 Zimbabwe Rhino Policy and Management Plan (1997) Cunningham et al. (1999) De Alessi (2000)
1993	Translocation and Reintroductions	Africa	Biological management and law enforcement action	Maintain productivity of established populations Creates additional populations with good growth prospects Improved chances of survival	Booth et al. (1984) Booth and Coetzee (1988) du Toit (2002) Emslie and Brooks (1999) du Toit (2002)
	Black Rhino Conservation Project Emergency Plan	Zimbabwe	Coordination, collaboration and program management	Led to the establishment of and operations within Intensive Protection Zones (IPZs) Appropriate management actions	Zimbabwe Rhino Policy and Management Plan of (1997) Cunningham et al. (1999) De Alessi (2000)
1994	Shoot to Kill Policy	SADC	Rhino protection	Militarization of conservation Effective deterrence to poachers The shoot‐to‐kill policy indicates that government considers poaching an act of war. Impressive elephant and rhino conservation	Milner‐Gulland et al. (1994) Duffy (2014) De Alessi (2000) Cheteni (2014) Mogomotsi and Madigele (2017)
2016	China Rhino and Rhino Product trade ban	Global	Regulated rhinoceros trading	Rhino parts to be used for medicine, scientific research, and cultural exchanges Rampant poaching and population decline Reopening illegal markets	Manley (2015) Crosta et al. (2017) Harvey et al. (2017) Whitfort (2019)

In 1985, the then Zimbabwe's Department of National Parks and Wild Life Management (now Zimbabwe Parks and Wildlife Management Authority) established Operation Stronghold, a system of patrols by armed game scouts in the Zambezi Valley (Wildlife, 1989; Zimbabwe Wildlife, 1989, 1989). Operation Stronghold's implementation was limited to armed antipoaching squads, game wardens, and Harare‐based park authorities, with some input by urban‐based, exclusively white‐controlled, conservation Non‐Governmental Organizations (NGO’s) (Hill, 1991; Wildlife, 1989; Zimbabwe Wildlife, 1989, 1989). In 1992 and 1993, Zimbabwe Black Rhinoceros Conservation Strategy and Black Rhinoceros Conservation Project Emergency Plan were formed, respectively (Alibhai & Jewell, 1994, 1994; Cunningham et al., 1999; De Alessi, 2000). These plans led to the establishment of and operations within Intensive Protection Zones (IPZs) and in private conservancies. The initial four IPZs were Chipinge Safari Area, Matopos National Park, Matusadona National Park, and Sinamatella Camp (Hwange National Park). This initiative was implemented concurrently with a full‐scale dehorning operation in an effort to deter poachers from killing the endangered species (Table 1). By 1994, poachers continued to kill rhinoceros despite radio collars (Figure 2), Communal Areas Management Program for Indigenous Resources (CAMPFIRE) (Murphree, 2009), conservancy programs (Chigonda, 2018), dehorning of hundreds of rhinoceros use of heavily protected animal sanctuaries, and a shoot‐to‐kill policy that left 178 suspected poachers and four game wardens dead (Fair Planet, 2019; Haysom, 2018; Milner‐Gulland et al., 1994).

The 1977 CITES ban failed because it artificially restricts supply in the face of persistent and growing demand (Bennett, 2015; Biggs et al., 2013; Hübschle, 2017a). By restricting the legal supply of rhinoceros horn (and associated supply‐side competition), the CITES trade ban may have increased the black market price of horn, thereby increasing the financial incentive to poach (Biggs et al., 2013; Hutton & Dickson, 2000) and caused the devaluation of live rhinoceros, resulting in many landowners opting out of rhinoceros conservation (Ferreira et al., 2014; Rubino & Pienaar, 2017).

In response to some of these threats, several conservation measures have been suggested and implemented with varying degrees of success in southern Africa. Despite these interventions, most rhinoceros populations occur in fairly small, isolated populations in conservancies, private game reserves, and intensive protection zones (Amin et al., 2006; du Toit, 2002; ZPWMA, 2015). Although there seems to be some consensus among conservationists regarding the main causes for the continued decline of these species, interventions toward reducing the threats seem fragmented and disjointed (Challender et al., 2015). Generally, most of the studies on rhinoceros conservation were explored in isolation without examining how these interventions could be integrated toward the development of holistic rhinoceros conservation management plans (Bending, 2018; Brandt et al., 2018; Harper et al., 2018). In this study, we attempt to fill this gap by providing an analysis of rhinoceros conservation threats, strategies, and opportunities for these species conservation. Specifically, the objectives of this study were to (i) determine the threats to rhinoceros conservation, (ii) examine the historical rhinoceros conservation strategies in Zimbabwe and Africa, and (iii) explore opportunities for enhanced and sustained rhinoceros conservation in Zimbabwe and Africa.

## 2 METHODS

### 2.1 Study area

This study focused on rhinoceros conservation perspectives in Zimbabwe and all countries in Africa with rhinoceros. For Zimbabwe, focus was on all rhinoceros regions including five (5) state‐owned and thirteen (13) private game sanctuaries.

### 2.2 Data collection and data analysis

Insights on two major themes pertaining to rhinoceros conservation were explored using holistic and historical perspectives (Mutanga et al., 2015; Powell, 2016). A holistic perspective implies an effort to shed light on the connections between and interactions of various phenomena in a greater whole (Gandiwa et al., 2014; Muboko et al., 2014). A historical perspective intends to uncover how events and phenomena in the past affect succeeding events and phenomena (Benbasat et al., 1987). A literature search from 1975 to 2020 was carried out using a predefined search protocol, involving a number of filters based on a set of keywords to balance search sensitivity with specificity (Pullin & Stewart, 2006). To achieve this, we searched Google Scholar and the Web of Science for journal articles using the terms ‘rhinoceros conservation,’ ‘rhinoceros conservation threats and challenges, ’African rhinoceros’, and ‘rhinoceros poaching,’ ‘rhinoceros illegal trade’, ‘rhinoceros conservation interventions’, ‘current conservation strategies’, ‘rhinoceros genetics’, ‘Zimbabwe’, ‘Africa’, ‘black and white rhinoceros’. We further used a “snowball” reference technique based on the sourced articles to extract older references that appeared to interrogate rhinoceros management and conservation threats. The collection of articles was fairly exhaustive though there could be other studies or articles, which fell outside the search parameters, or were published in lesser known volumes, or may not have been cited by later works. Of the 252 articles sourced, 193 articles that were more relevant to key themes on rhinoceros conservation in this study were finally used. Of the 193 articles used, 27 articles (16 before the year 2020 and 11 in the year 2020) mentioned marginalized communities and how they either influence or influenced by rhino conservation. The literature, which contained the aforementioned keywords in the abstract, was included in the analysis list, while the rest of the literature was discarded (Naderifar et al., 2017). The main threats, historical strategies, and rhinoceros opportunities for rhinoceros conservation were categorized into themes, which enabled us to use inductive content analysis approach. Inductive content analysis is a qualitative method of content analysis that researchers use to develop theory and identify themes by studying documents, recordings, and other printed and verbal material. The inductive content analysis approach allowed us to derive themes from interpreting each article and later grouping these into each of the identified themes. The themes enabled us to analyze the threats and challenges associated with rhinoceros conservation in Zimbabwe and in Africa as a whole.

## 3 RESULTS

### 3.1 Threats to rhinoceros conservation in Zimbabwe and Africa

The major local, regional, and international threats to rhinoceros conservation identified include illegal harvesting, illegal trade, habitat fragmentation and loss, and marginalization of local people, which leads to small and isolated populations vulnerable to genetic, demographic, and environmental stochasticity.

#### 3.1.1 Illegal harvesting

**FIGURE 1:**
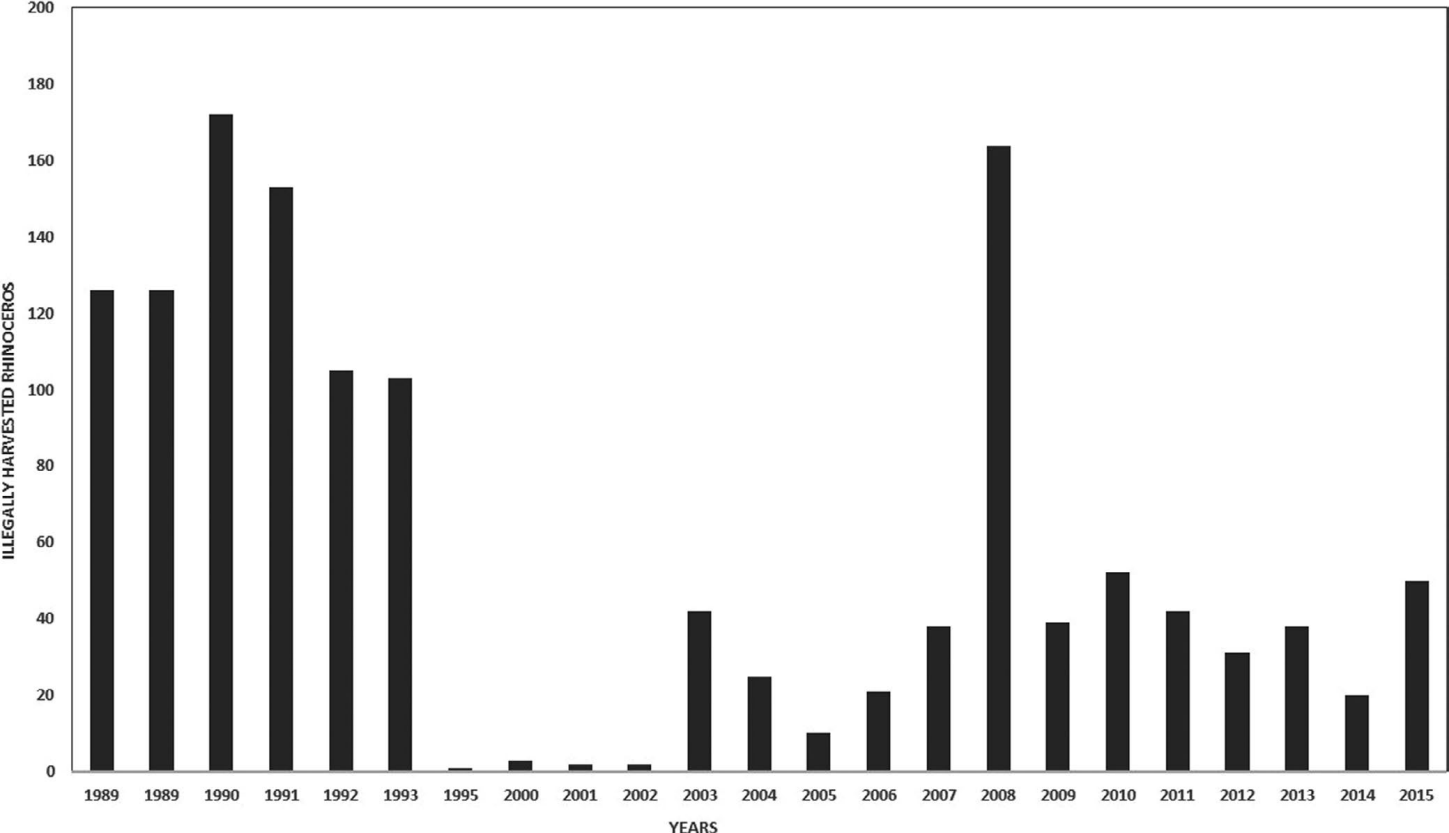
Number of illegally harvested rhinoceros in Zimbabwe for the period 1989–2015. Sources: Lindsey and Taylor (2011), Milliken and Shaw (2012), World Wildlife Fund Zimbabwe and Zimbabwe Parks and Wildlife Management Authority

Illegal harvesting is motivated by the international demand for rhinoceros horns for medicinal uses and perceived sociocultural symbolic values (Hübschle, 2017a,2017b; Smith, 2018, 2018). With reference to Zimbabwe, over 1,000 black rhinoceros were known to occur in several protected areas in the mid‐1980s (Biggs et al., 2013; Booth et al., 1984). This includes what was then known as the largest single black rhinoceros population in the world, which occurred in the Mid‐Zambezi Valley (De Alessi, 2000). Amidst the gloom of crumbling world economies, global health systems, and the new COVID‐19 pandemic (Saeed et al., 2020), one positive effect of the pandemic is that it has drawn the attention of the world to the global problem of illegal harvesting and illegal wildlife trade (Beirne, 2020; Corlett et al., 2020; Roth, 2020). However, by the year 1994, the black rhinoceros population in Zimbabwe had dropped to less than 300, mainly due to increased poaching pressure (De Alessi, 2000) (Figure 1).

**FIGURE 2:**
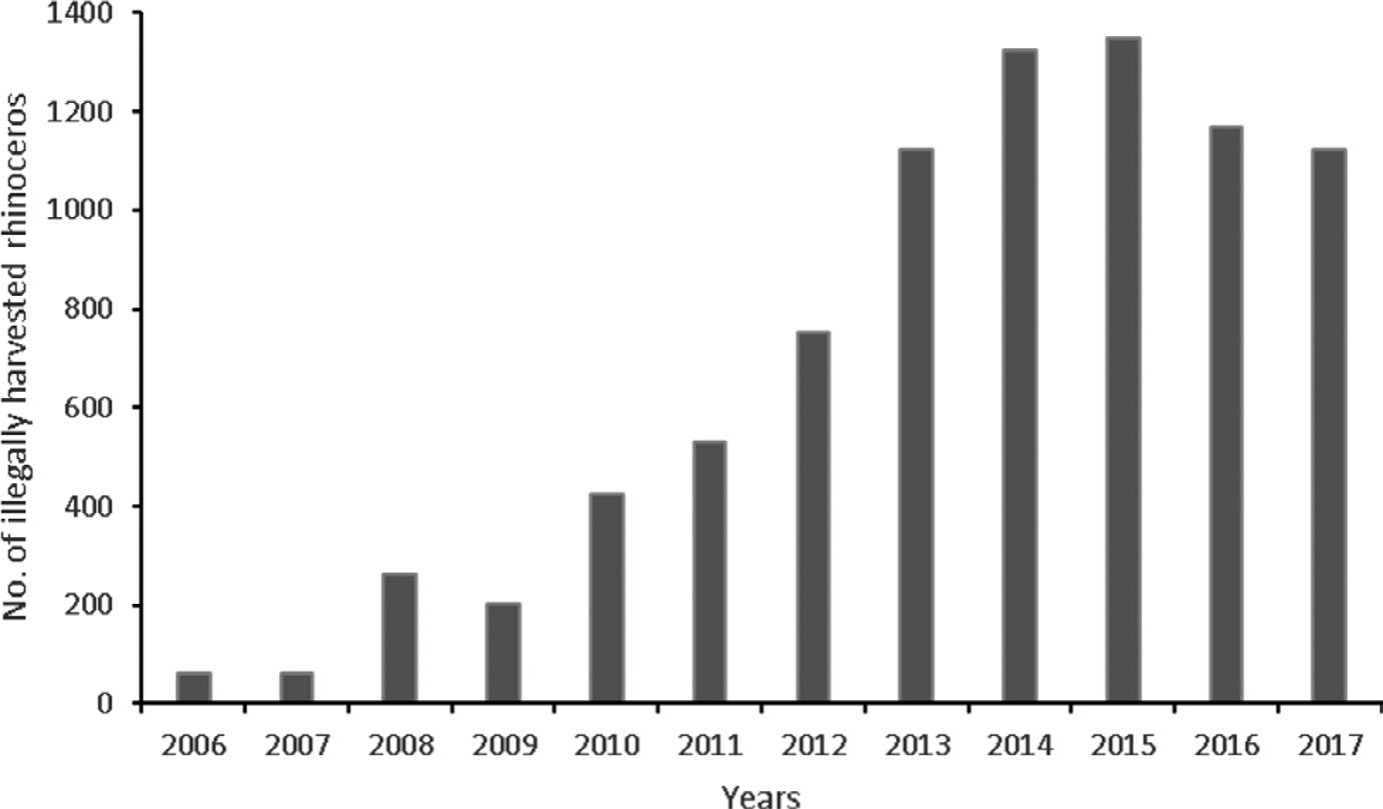
Reported number of illegally harvested rhinoceros in Africa for the years 2006–2017 (Save the Rhinoceros, 2018)

Though poaching in Zimbabwe was a problem prior to the year 2008, the levels were considered to be fairly low. However, there was a continued increase in the number of rhinoceros poached annually in Africa from the year 2008 to 2015, with an unprecedented high number of 1,342 rhinoceros poached in the year 2015 (Figure 2). The illegal killing of rhinoceros for international trade of the horn has been reported as the greatest threat to the persistence of this endangered species (Annecke & Masubelele, 2016; Standley & Emslie, 2013). Evidence shows that the continued existence of illegal rhinoceros horn markets and the increasingly high illegal market prices, due to a surge in demand, remains a key threat (Emslie, 2013; Ferreira et al., 2018). These unprecedented levels of poaching compromise the viability of rhinoceros population in the wild and require strategic interventions to ameliorate this scourge trade (Duffy, 2014; Emslie, 2013; Emslie et al., 2016; Ferreira et al., 2018).

#### 3.1.2 Illegal wildlife trade

The increase in rhinoceros poaching could be attributed to the purported widespread demand and increase in the value of the horn in Vietnam and other countries (Dang & Nielsen, 2018). This trend is not surprising given that illegal wildlife trade is estimated to be worth several billions of dollars annually (Hanley et al., 2018; Hübschle, 2016; Smith, 2018, 2018). This ready market and demand for wildlife products makes it comparable to other organized crime and illegal trade commodity syndicates such as drugs and firearms (Mogomotsi & Madigele, 2017). The poaching crisis for rhinoceros horn remains unabated due to the existence of criminal syndicates, which offer economic incentives to perpetrators. Most perpetrators of illegal wildlife products are backed by international criminal syndicates, which make it profitable to engage in poaching and trafficking of wildlife products (Challender & MacMillan, 2014; Ferreira et al., 2018; Vandome & Vines, 2018).

The issue of legalization is a complex one due to the fact that the rhinoceros market is not well‐known. As the illegal trade has grown, revenue from sales of live rhinoceros has decreased, and the increased cost of protecting the rhinoceros is substantial. However, if intelligence is properly collected and analyzed, it can provide reliable information about sales, markets, consumer demand, and, most importantly, the activities of the criminal networks. In Africa and Asia, there is corruption among government and conservation officials who are sometimes bribed to turn a blind eye to illegal transactions and shipments of rhinoceros horn, or are even more directly involved in the crimes (Challender et al., 2015; Duffy & Humphreys, 2014; Haas & Ferreira, 2016). Poached rhinoceros horn leaves Africa mostly through Mozambique or South Africa, mainly destined for Vietnam en route to China (Montesh, 2013; Smith, 2018, 2018; Veríssimo et al., 2012). Other countries in eastern Asia, notably Laos and Myanmar, have become involved in the cross‐border trade in rhinoceros horn, mainly to meet Chinese demand (Duffy, 2014; Haas & Ferreira, 2016). Traffickers take advantage of weaknesses in governance and detection systems all along the chain. Rhinoceros horn from poached animals in Africa passes through the hands of several traders operating on different levels before leaving the continent en route to eastern Asia. Enforcement efforts against general wildlife trade in developing countries have generally been unstructured and underfunded resulting in the proliferation of illegal wildlife trade (Challender & MacMillan, 2014; Gandiwa et al., 2013a, 2013b; Holden et al., 2019). In some cases, weak law enforcement efforts in some protected areas have resulted in pseudo‐open‐access resource systems, which expose rhinoceros to more poaching (Challender & MacMillan, 2014; Ogutu et al., 2016). Increased poaching pressures have resulted in escalating costs of rhino ownership and conservation, in particular, security costs related to protection of rhinoceros from poaching (Collins et al., 2017; Rubino & Pienaar, 2018). In some countries, weak enforcement efforts emanate from inadequate funding of conservation law enforcement agencies, thus exposing African rhinoceros to poaching risk (Coetzee, 2012; Ferreira et al., 2018; Gandiwa et al., 2013a, 2013b).

Much concern is that rural communities, accustomed to employment with hunting outfitters, may relapse to poaching for meat and animal parts for the black market as hunting industry layoffs build due to COVID‐19 pandemic (Lendelvo et al., 2020; Neupane, 2020; Zela, 2020). The outbreak of COVID‐19 pandemic has also brought to the fore issues of food safety and standards and public health associated with illegal wildlife trade (Duonamou et al., 2020). In China, the pandemic has provided impetus for law reform (You, 2020). Corruption is believed to be one of the key drivers of illegal trafficking and trade in wildlife (Coetzee, 2012; Smith et al., 2015). While the trade in protected species is highly regulated under international law, the world market in illegal wildlife products continues to grow and thrive (Borzée et al., 2020; Evans et al., 2020; You, 2020). Nonetheless, addressing corruption and organized crime is critical in combating international wildlife trade.

#### 3.1.3 Habitat fragmentation and cascading effects

The underlying cause of virtually all recent and ongoing declines of mammal species is the growth of human populations and the associated impacts (Cardillo et al., 2004; Gill et al., 1996). Habitat loss and modification are adversely affecting the conservation of rhinoceros not only in Zimbabwe but also in Africa as a whole (Baudron et al., 2011; Child, 1995). For example, parts of Save Valley Conservancy and Midlands Black Rhinoceros Conservancy in Zimbabwe were invaded by local people during the fast track land reform phase in early 2000s (Chibisa et al., 2010). Concerns have been raised regarding the sustainability of two conflicting land uses and their potential impacts on species persistence in these conservation areas (Chibisa et al., 2010; Ndlovu, 2015; Scoones et al., 2011). To some extent, these invasions and land transformations exposed wildlife to poaching. For example, Bubiana Conservancy had a key population of over 100 rhinoceros, but this number declined due to habitat loss and poaching caused by land invasion (Chibisa et al., 2010; De Alessi, 2000; Wolmer, 2005). Habitat fragmentation can threaten population persistence by diminishing the size of habitat patches, isolating patches of habitat, and creating edge effects. Fragmentation inevitably leads to the juxtaposition of qualitatively different habitats, flow of materials, and individuals between them can indirectly exert profound influences on within‐fragment communities (Baudron et al., 2011; Lander & Brunson, 2018). Protected areas and buffer zones continue to suffer from human encroachment, and grazing by domestic livestock is causing serious damage in some localities (Strayer et al., 2003; Wang et al., 2006). Consequently, the suitability of rhinoceros habitats is inevitably compromised. In some cases, alien invasive plant species are also invading some of the grasslands on which the rhinoceros depend, dominating and destroying indigenous palatable vegetation (Bhatta et al., 2020; Lahkar et al., 2011; Rai & Singh, 2020). Humans meddling with habitats during the pandemic are also imperiling some species. The loss of habitats, the modification of natural environments, and more generally the decline in biodiversity are all factors in the spread of emerging infectious diseases (Ahmand et al., 2020; Wilkinson et al., 2018).

Although habitat fragmentation has been documented to affect viability of wildlife populations, this has not been the case with rhinos, which are mostly found in secure areas, for example, Intensive Protection Zones or private areas or parks, which are not prone to fragmentation or encroachment (Ndlovu, 2015). Nonetheless, this could be a challenge in countries where land tenure systems are contested and unstable. Habitat disturbances generally lead to fragmentation and isolation of wildlife populations (Fahrig et al., 2019; Fletcher et al., 2018). Here, we argue that in cases with habitat fragmentation, viability, and persistence of the small, isolated rhinoceros populations would be compromised. This challenge is exacerbated and more pronounced in cases where fragmentation occurs in combination with illegal harvesting, which magnify the impacts of small, isolated rhinoceros populations. The persistence of the resultant small meta‐populations is compromised due to their vulnerability to demographic (Brandt et al., 2018; Hogg et al., 2017), environmental (Child, 2012a,2012b; Ferreira de Souza Dias, 2013; Ndlovu, 2015), and genetic stochastic events (Frankham, 2016; Hübschle, 2017a,2017b; Mays et al., 2018; Ralls et al., 2018). One critical genetic stochastic process that affects viability of rhinoceros in the wild is inbreeding depression, which results in loss of genetic variation in a meta‐population (Pizzi et al., 2013; Whiteley et al., 2015). This loss of genetic variation compromises reproductive success of individuals and reduces adaptation to environmental pressures such as extreme weather events, diseases, pests, and parasites (Falk & Holsinger, 1991; Frankham, 1995). Consequently, there is dire need for strategic and holistic managed initiatives to reduce the probability of extinction in wild populations emanating from the stochastic processes.

#### 3.1.4 Marginalization of local people in conservation

Local people are pushed to the periphery of rhinoceros protected areas where they do not benefit from conservation, but they are confronted with a serious challenge of having to contend with conflict with wildlife (Fynn, 2020). It is argued that the current disruptive regime in the form of “the war on poaching” and the displacement of communities from parks and buffer zones contribute to the social reproduction of historical inequalities, stigmatization, and alienation of communities, who under different circumstances and framing might be agents of change and disruptors of illegal wildlife trafficking (Fenio, 2014; Hübschle, 2017a; Witter & Satterfield, 2019). There seems to be more antagonism between communities and conservation authorities because of high incidences of human–wildlife conflict, limited employment opportunities, and economic incentives at household level (Fenio, 2014).

Conservation actors, policymakers’ donors, and communities should move beyond the premise of the fortress conservation paradigm, which assumes conflictual relationships between rural communities and wildlife (Haysom, 2018; Stoett, 2019). If local communities remain on the margins of protected areas and are excluded from the economic benefits of conservation, they will fail to support the conservation drive, or even take to poaching. The sociopolitical, historical context, and continued marginalization are significant factors leading to poaching decisions at the grassroots level (Hübschle, 2017a).

The degree to which decentralization and devolution can take place depends very much on political priorities and influences, as well as the capacity of all governance actors (Biggs et al., 2017; Mutanga et al., 2015). This requires a delicate balance in order to accommodate broad stakeholder priorities and capabilities. The shame of not being able to provide for their families’ emasculation, stress, disempowerment, and anger is motivating local communities to poach (Hübschle, 2017a).

#### 3.1.5 External shocks in biodiversity conservation

Complex ecosystems possessing sufficient scale and original biodiversity typically exhibit resilience to natural shocks as they remain within a favorable basin of attraction (Barrett et al., 2011). External shocks in biodiversity conservation are difficult to deal with considering that they affect both environmental and social aspects of the system, for example, the global financial recessions (Kideghesho & Msuya, 2012) and pandemics such as the COVID‐19. These pandemics directly and indirectly affect revenue toward conservation financing, law enforcement, and ultimate species conservation efforts (Zela, 2020). COVID‐19 has had huge impacts on multiple industries and sectors, not just wildlife protection. Given extremely limited resources, governments are likely to abandon wildlife protection in the short to medium term and redirect resources to humanitarian considerations (Evans et al., 2020). Some critical wildlife conservation programs are facing funding cuts due to revenue shortfalls occasioned by COVID‐19 disruptions (Corlett et al., 2020; Evans et al., 2020; Gössling et al., 2020). The COVID‐19 pandemic anthropause, which is the global‐scale, temporary slowdown in human activity, is likely to have a profound impact on other species. To seize this opportunity, there is need for collaborative initiatives to research the effect of the anthropause. In the post‐COVID‐19 pandemic era, mobilizing budgets for biodiversity could be harder than ever before. With less revenue coming from tourists and funders into the sector conservation, efforts have been crippled (Gössling et al., 2020; Higgins‐Desbiolles, 2020; Zela, 2020). Under these circumstances, it is most likely that cases of poaching and illegal wildlife trade will increase.

## 4 DISCUSSION

### 4.1 Opportunities for rhinoceros conservation

Several rhinoceros conservation strategies have been suggested and implemented in an attempt to reduce poaching and enhance conservation outcomes. These opportunities usher in dynamic and holistic options for rhinoceros conservation. However, their adoption and success vary from one location to the other due to different operating environments and enabling factors (Codron et al., 2007).

### 4.2 Demand reduction awareness and campaigns

The supply chain of wildlife and wildlife products from source areas, and transportation through local, national, and international networks to distant markets allow for natural spillover and spread. Due to high demand for wildlife products, reducing illegal wildlife trade remains an elusive undertaking (Baker et al., 2018; Beirne, 2020; Borzée et al., 2020). Public education campaigns should not only tell people about how the wildlife trade (both legal and illegal) harms endangered species, but also its public health implications giving example to COVID‐19 pandemic (Beirne, 2020; Bending, 2018; Biggs et al., 2017; Challender et al., 2015). While poaching and illegal animal trading are not a new phenomenon, the COVID‐19 health emergency has exacerbated the problem. With the focus on demand management, it is envisaged that there would be a reduction in the market value of illegal wildlife products through getting consumers to voluntarily change their purchasing behavior (Ayling, 2016; Wallen & Daut, 2018).

However, demand reduction efforts might hit a brick wall as countries such as South Africa have been given a green light to increase black rhinoceros hunting quotas. This could increase the market value of rhinoceros and rhinoceros products from Zimbabwe and other Southern Africa. There is need for adequate understanding of demand and the powerful market forces in consumer countries that overwhelm enforcement efforts. Strengthening protected area networks, rhino habitat preservation, eradication of the illegal trade in rhino horn, crack down on poaching of rhinos, and conservation awareness are the tasks at hand. Demand reduction awareness and campaigns are high‐order opportunities, which are supported and enabled by international funding boards and intergovernmental collaborations (Codron et al., 2007). Table 2 presents summary of threats, strategies, opportunities, and enablers in rhinoceros conservation.

**TABLE 2:** Summary of threats, strategies, and opportunities in rhinoceros conservation

Scale	Threat	Strategy	Opportunity	Enablers
International	Illegal international wildlife trade Illegal harvesting External shocks including global financial recessions and pandemics, that is, COVID‐19, Ebola	Education and awareness campaigns Enhanced capacity building in rhino conservation Diversified and sustained funding mechanisms Use of adaptive safety nets, for example, Global Safety Net Coordinated international conservation partnerships for cooperation	International demand reduction awareness and campaigns Enhanced law enforcement initiatives, crime detection, and forensics Wildlife or conservation cryptocurrency eco‐tax Potential conservation credits and eco‐labeling	Inclusive and sustained partnerships Sustainable funding options Donors International community
Regional	Illegal harvesting Illegal wildlife trade	Regional corporation and partnerships on law enforcement, crime detection, and tracking Transboundary conservation efforts	Enhanced law enforcement initiatives, crime detection, and forensics Facilitation of formal regional protocols and use of wildlife enforcement monitoring system (WEMS) initiatives.	Collaboration Donors and conservation financing partners Political goodwill
National	Habitat fragmentation and loss Poaching and cascading effects of small populations, for example, inbreeding depression Weak policy and institutional settings and corruption	Strengthened habitat protection and management initiatives Strategic dehorning programs Re‐introduction programs Innovative systems and surveillance equipment for real‐time monitoring	Strategic science‐based meta‐population management Enhanced law enforcement initiatives, crime detection, and forensics Strengthened and transformative policies and institutional frameworks	Conservation partnership and collaborations Political goodwill Financial resources Good corporate governance
Local	Disgruntled and marginalized local people in conservation areas Weak policy and legal frameworks	Environmental education and awareness campaigns Local community participation and involvement Enhancing livelihoods of local communities	Community‐led demand reduction campaigns Capacity building and conservation incentives for vulnerable and marginalized communities Women empowerment initiatives	Conservation partnership and collaborations Supportive local communities Financial resources

### 4.3 Community Involvement and Participation

With the advent of COVID‐19, the lure of getting a few dollars to sustain one's family may prove irresistible for some community members (Zela, 2020). Under these circumstances, it is most likely that cases of poaching and illegal wildlife trade will increase (Roth, 2020). Community‐based initiatives must be given the support they need to deliver incomes to local people through legal wildlife utilization, incomes that are crucial in alleviating poverty (Bassett, 2005; Duffy et al., 2013; Duffy & St John, 2013; Taylor et al., 2016). This support should include the right for indigenous people and local communities to be consulted as equal partners in rhinoceros conservation and a bottom‐up negotiating approach should be used. Some properties have community projects, for example, Lowveld Rhino Trust (Lowveld Community Trust Program). Provision of alternative livelihoods, increasing incentives for stewardship, mitigating against human–wildlife conflict, helping COVID‐19 pandemic victims, and strengthening disincentives for poaching (Biggs et al., 2019; Holden et al., 2019; Taylor et al., 2016) are all elements of a comprehensive response to illegal wildlife trade (Biggs et al., 2017, 2019; Holden et al., 2019; Hübschle, 2017b; Quesada, 2019). There is need to educate local communities about COVID‐19 and hand out sanitary supplies to help protect against its spread (Lendelvo et al., 2020).

Critically, in developing community conservation packages, we must look beyond compensation payments based on opportunity costs, which may not always incentivize conservation and look to create prosperity locally from managing the conservation of high‐value wildlife (Table 2) (Challender & MacMillan, 2014). Enhance incentives for rhino conservation through public–private community partnerships (Di Minin et al., 2015; Grooten & Almond, 2018; Janssens & Trouwborst, 2018), especially with this advent of the COVID‐19 pandemic. However, at a time when engaging with local stakeholders is more important than ever, COVID‐19 makes this much harder. Yet without continued communication between local people, reserves, and conservancies, the risk of poaching could increase (Fynn, 2020; Roth, 2020).

Law enforcement efforts can be supported by members of the public and communities through provision of secure mechanisms to anonymously report wildlife and biodiversity crimes (Codron et al., 2007; Mogomotsi & Madigele, 2017). Innovative applications such as Wild Information and Landscape Database (WILD), Project Poacher, and Spatial Monitoring and Reporting Tool (SMART) among other mobile applications could help thwart poachers in remote areas and beyond. Environmental Education Programs (Ali et al., 1999; Coltman et al., 1999; Keane et al., 2008) with clear objectives and measurable impacts should be established and run for schools and communities surrounding key rhino populations (Hungerford et al., 1980; Short, 2009). Examples of rhinoceros properties encouraging EEPs in Zimbabwe are Imire Game Park and Lowveld Rhino Community Trust (Zazu, 2007).

The current rhinoceros control paradigm and associated conservation policies are aimed at controlling poachers and advancing security and other antipoaching measures to disrupt wildlife trafficking networks (Ferreira et al., 2015; Roe & Booker, 2019; White, 2014). Securitization and militarization, however, close down pathways for community empowerment (Fynn, 2020). Devolving power and benefits to local communities will enable local communities to acquire full responsibility for antipoaching operations, which they are much better to do than external agencies who do not have the social networks and local knowledge needed to effectively perform oversight functions in the local area (Biggs et al., 2017; Fynn, 2020; Roe & Booker, 2019). Understanding and working with local cultures and beliefs can create significant opportunities for conservation. Although the Parties to CITES have recently reiterated the importance of local community livelihoods in regulating trade, with the adoption of Resolution 16.6 (CITES and livelihoods), it is essential that this policy is converted into action with livelihoods given greater attention in listing decisions, implementation, and funding (Challender & MacMillan, 2014). Governments, community partnerships, and donors as chief enablers should fully support and capacitate communities living with rhinos (Codron et al., 2007). Strategies that harness local values and institutions to promote pro‐rhino behavior are likely to be more effective in the long term since they seek to change the attitudes, intentions, and ultimately behavior of the people most likely to be exposed to and tempted toward engaging in poaching activity (Baker et al., 2018; Ferreira et al., 2018; Fynn, 2020). Communities need to diversify the key rhino‐based tourism, local tourism market, explore more adventure tourism, include agricultural possibilities, and expand sustainable‐use options(Roe, 2015; Roe & Booker, 2019). Post‐COVID‐19 indigenous models based on natural resource governance, traditional knowledge, and food sovereignty should be developed (Everingham & Chassagne, 2020; Samarathunga, 2020). Efforts should be made to develop alternative livelihoods and economic models around core wildlife areas and species that are less dependent on extractive use of wildlife.

### 4.4 Enhanced law enforcement initiatives, crime detection, and forensics

It is critical that monitoring, protection, and intelligence activities continue uninterrupted to ensure the safety of rhinos in southern Africa, especially in this COVID‐19 era. The involvement of transnational organized criminal syndicates in horn trafficking has been met with increased law enforcement efforts to apprehend, prosecute, and sentence traffickers and poachers with the aim of reducing poaching (Challender & MacMillan, 2014; Harper et al., 2018; Veríssimo et al., 2012). Rhinoceros poaching is known to be more sophisticated and syndicate‐driven, which reduces effectiveness of traditional law enforcement measures. To augment traditional law enforcement initiatives, advanced technology and innovative systems such as seismic sensors, satellites, drones, field closed‐circuit television camera (CCTV’s), and sniffer dogs can be used to aid in the monitoring and protection of black and white rhinoceros in Zimbabwe (Annecke & Masubelele, 2016; Bending, 2018; Duffy, 2014). However, these interventions require a holistic approach, which is strengthened by international support (Codron et al., 2007), strategic and effective legal and policy frameworks, and augmenting law enforcement frameworks (Veríssimo et al., 2012). A critical mass of committed local people working hand in hand with law enforcement would be a formidable barrier to would‐be poachers and is likely the only measure that will stand up to the relatively huge financial rewards that poaching currently provides. The international operating procedures and support should also cascade to local scale. Policy and legal frameworks related to law enforcement should therefore be tailored to factor in the underlying drivers of poaching and trade as well as the complex social, cultural, and economic nature of the phenomenon (Biggs et al., 2017; Moore et al., 2006).

In some cases, law enforcement efforts ought to strengthen investigation and wildlife species identification capacity through forensic techniques. Possible approaches that may be up‐scaled include footprints analysis at the crime sites, morphological study of the species, serological methodology, and molecular techniques (Harper et al., 2018; Iyengar, 2014; Mays et al., 2018). Although deoxyribonucleic acid (DNA)‐based protocols on crime scene carcasses to link confiscated evidence to specific poaching incidents for support of criminal investigations and evidence gathering have been developed over the years (Brandt et al., 2018; Frankham, 2016; Frankham et al., 2014; Harper et al., 2013), capacity remains low in most African countries. Efforts should be directed toward funding and technical support toward the establishment, capacitation, and accreditation of wildlife forensics laboratories, to guarantee credibility of forensic evidence (Iyengar, 2014). Though these wildlife forensic approaches are useful for evidence gathering, several challenges are associated with their applications. Nonavailability of species‐specific antibodies in serological analysis, undetectable footprint and erosions by the other animals in footprint analysis, requirement of samples in well‐preserved form in microscopic analysis, lack of taxonomic keys, and animal monographs have been identified as some of the challenges (Brandt et al., 2018; Harper et al., 2018; Haysom, 2018). Government agencies and wildlife conservationists are aware of the need to collaborate across borders, and if intelligence is shared and acted upon, it is one of the most cost‐effective tools for law enforcement (Haas & Ferreira, 2016). For example, the Southern African Development Community (SADC) Law Enforcement and Antipoaching Strategy: 2015–2020 (SADC LEAP) was crafted to address poaching in the region. The SADC LEAP has five strategic areas: (1) enhancement of legislation and judicial processes; (2) minimization of wildlife crime and illegal trade; (3) integration of people and nature; (4) sustainable trade and use of natural resources; and (5) improvement and strengthening of field protection (Secretariat, 2017). A regional SADC Wildlife Crime Prevention and Coordination Unit (Table 2) could actively facilitate the implementation and evaluation of the strategy (Codron et al., 2007). In this regard, more efforts could be made toward refining all these techniques to guarantee their universal application of crime investigations and wildlife forensics.

### 4.5 Enhanced science‐based meta‐population management

In meta‐populations, systems of local populations in suitable, discrete habitat patches interact via dispersal of individuals moving in the matrix. To mitigate inbreeding and resultant loss of genetic diversity in small isolated rhinoceros populations, strategic genetic‐based meta‐population management is requisite (Ali et al., 1999; Mackey et al., 2006; Ouborg et al., 2010). In areas with spatially constrained or residual rhinoceros populations that occur as small and somewhat isolated with limited dispersal capabilities, management of breeding individuals to promote gene flow and reproductive success is critical (De Alessi, 2000; Martin, 1993; ZPWMA, 2015). Genetic‐based meta‐population management requires national and local management agencies to have a sound knowledge on the genetic diversity and level of relatedness among individuals in these populations (Iyengar, 2014; Whiteley et al., 2015). To enhance these strategies, research and monitoring initiatives toward identification of site‐based threats in relation to ecosystem integrity and habitat suitability for rhinoceros population viability could be promoted in rhinoceros (Biggs et al., 2015; Nichols & Williams, 2006). More so, research findings on strategic meta‐population‐based initiatives could be timely shared among stakeholders, scientists, policymakers, government, and concerned politicians (Codron et al., 2007) to bridge the gap between science and policy for effective rhinoceros conservation in Africa. However, biological fieldwork must be possible despite restrictions and funding for follow‐up studies, which is required to compare data from before, during, and after the anthropause (Rutz et al., 2020).

### 4.6 External shocks in rhinoceros conservation

Restrictions on international travel due to COVID‐19 pandemic have seen a crash in the long‐haul tourism market and conservation hunting that has been the cash cow for many African range states (Gössling et al., 2020; Higgins‐Desbiolles, 2020; Neupane, 2020). COVID‐19 is pushing many tourism operations to close, unemployment to rise, conservation organizations to the brink of failure, and communities to be skeptical about the value of wildlife as a viable form of land use. The COVID‐19 lockdown could even be catastrophic for some endangered species. In Africa, iconic species such as the rhino rely on protected areas and armed guards, all funded by tourism money that is evaporated and may not fully return for two years (Borzée et al., 2020; Neupane, 2020). That becomes a real issue in terms of guards being there both being allowed to be there with social distancing, but also in terms of them getting paid to do the work. Out‐of‐the‐box thinking is needed to come up with innovative funding mechanisms for conservation endangered species (Gutman & Davidson, 2007; Whitelaw et al., 2014), especially in this COVID‐19 era. Reduced human mobility during the pandemic will reveal critical aspects of our impact on animals, providing important guidance on how best to share space on this planet. The mechanisms can vary across levels from climate taxes, wildlife tax (Connelly & Brown, 1994; Godsey, 2018) at national level, conservation cryptocurrency (Chesney, 2019; Mofokeng & Fatima, 2018), and donations to issues of potential wildlife credits similar to carbon trading to cover the needs to fund conservation in cases of global shocks. For example, Save the Rhino has launched a Rhino COVID‐19 Crisis appeal, supporting different programs in Africa. The appeal is not only giving a voice to the stories that many would otherwise not hear: the impact on a ranger's family in lockdown and the importance of updating health and safety procedures for antipoaching teams. It is also raising funds to support programs in this time of crisis, helping to fund basic but essential items, such as unexpected costs from the virus and vehicle maintenance, that all cost money, which is in short supply (Lindsey et al., 2020; Waithaka, 2020).

Governments can use and rely on adaptive safety nets that use existing social protection schemes and can quickly expand by increasing the number of beneficiaries and the sums transferred to them (Cardinale et al., 2012; Rahmato, 2013). It is an efficient way to help people and biodiversity after a major shock. Governments could be guided to identify viable biodiversity investment areas in their recovery programs and make use of them (Ervin et al., 2010). More optimistically, education and research in ecology, conservation, and environmental studies may appear more attractive and meaningful to young people who have been alerted to the global environmental crisis by this pandemic and made aware of the links between biodiversity conservation and human well‐being (Corlett et al., 2020). Eliminating some of the COVID‐19 pandemic impacts altogether will be difficult in the short to medium term. However, valuable lessons for conservation have been drawn from these extreme events and external shocks as outlined by Lindsey et al. (2020). Coordinated global wildlife research during the anthropause will make contributions that go well beyond informing conservation science and it will challenge humanity to reconsider our future on earth.

## 5 CONCLUSION

This study established the following important threats to rhinoceros conservation; inadequate funding and law enforcement; weak policy and legal frameworks; and disgruntled and marginalized communities who antagonize conservation efforts, illegal harvesting and international trade, and habitat fragmentation and loss. The current rhino management strategies have over the years been framed with the following aspects in consideration: biological management and monitoring, effective protection and law enforcement, socioeconomic sustainability, and coordination, collaboration, and program management. Biodiversity conservation external shocks such as pandemics are unpredictable and remain a cause for concern in biodiversity conservation. For instance, COVID‐19 pandemic and global financial recession directly and indirectly affect revenue channeled to species conservation efforts. The absence of tourists, rangers, and conservationists has made poaching easier in many protected areas, while the lack of tourists increased financial pressure. It is essential to use all the available opportunities and enhance collaborative conservation efforts in species conservation strategies such as (i) embracing strategic scientific‐based meta‐population management to improve fitness and reproductive success of individuals, (ii) a well‐coordinated stakeholder and community involvement, (iii) enhancing demand reduction campaigns with local community conservation groups, (iv) enhancing monitoring and law enforcement capabilities by adopting the use of Wildlife Enforcement Monitoring System (WEMS) and facilitation of formal regional protocols, and (v) customizing intelligence mechanisms and appropriate technologies in crime detection and wildlife forensic.

## CONFLICT OF INTEREST

The authors declare that they have no financial or personal relationship(s) that may have inappropriately influenced them in writing this article.

## AUTHOR CONTRIBUTION

**Admire Chanyandura:** Conceptualization (lead); Formal analysis (equal); Methodology (lead); Resources (lead); Writing‐original draft (lead); Writing‐review & editing (equal). **Victor K. Muposhi:** Conceptualization (supporting); Formal analysis (supporting); Methodology (equal); Project administration (equal); Resources (equal); Supervision (lead); Validation (equal); Visualization (equal); Writing‐review & editing (equal). **Edson Gandiwa:** Conceptualization (equal); Formal analysis (equal); Methodology (equal); Project administration (equal); Resources (equal); Supervision (equal); Visualization (equal); Writing‐review & editing (equal). **Never Muboko:** Formal analysis (equal); Methodology (supporting); Validation (equal); Visualization (equal); Writing‐review & editing (equal).

## Data Availability

All the journals, reports, and books used for the review are freely and publicly available on Google (https://www.google.com/) and Google Scholar (https://scholar.google.com/) search engines. DOI accession number from Dryad: https://doi.org/10.5061/dryad.kprr4xh49; https://datadryad.org/stash/resources/111064/review
